# Treatment of biliary tract cancer - essentials for clinical practice

**DOI:** 10.3389/fonc.2026.1750154

**Published:** 2026-03-10

**Authors:** Marian Liberko, Renata Soumarova

**Affiliations:** 1 Department of Oncology, Third Faculty of Medicine, Charles University and FNKV, Prague, Czechia; 2 Third Faculty of Medicine, Charles University, Prague, Czechia

**Keywords:** biliary tract tumours, immunotherapy, NGS testing, systemic treatment, targeted therapy

## Abstract

Biliary tract tumours represent a serious medical problem due to their high mortality rate. In early stages, patients are indicated for postoperative therapy after potentially curative surgery in order to reduce the risk of recurrence and prolong survival. Most patients with bile duct tumours are diagnosed at a locoregionally advanced and/or metastatic stage. The standard treatment for these patients is systemic therapy, now in combination with immunotherapy. Deepening knowledge of the molecular biology of this disease allows for the selection of treatment tailored to the individual patient based on the presence of specific targetable alterations in some patients. The article provides an overview of current treatment options for this disease across all stages.

## 1 Introduction

Biliary tract tumours account for approximately <1% of all tumours. Cholangiocellular carcinoma (CCA) is the second most common liver tumour after hepatocellular carcinoma and accounts for approximately 10%–15% of primary liver tumours (1). From an anatomical point of view, biliary tract tumours can be divided into intrahepatic (iCCA), perihilar (pCCA), and distal (dCCA). In the vast majority of cases, they are adenocarcinomas.

The aetiology of CCA is multifactorial and depends to a certain extent on specific risk factors in individual regions. A common risk factor for bile duct tumours is chronic inflammation of the bile duct epithelium. There is a known association between the occurrence of cirrhosis, hepatitis B virus (HBV) and hepatitis C virus (HCV) infections, and CCA. Diabetes mellitus, obesity, and metabolic syndrome are also significant risk factors for CCA. In the case of gallbladder tumours, chronic cholecystitis, gallstones, porcelain gallbladder, and gallbladder polyps are generally accepted risk factors (1). Specifically, primary sclerosing cholangitis is well known factor for hilar cholangiocarcinoma (2).

The incidence of CCA varies between regions, but globally, there is a clear trend towards an increase in the incidence of iCCA and a decrease in the incidence of dCCA. Increase in the incidence of iCCA may be in part due to the declining rate of cancer of unknown primary, which were previously incorrectly labelled as intrahepatic cholangiocarcinoma. Due to the asymptomatic early stages of iCCA, this disease is diagnosed in the vast majority of cases as locally advanced or metastatic, whereas in dCCA, where obstructive jaundice is a common first symptom, a higher percentage of patients are diagnosed at a non-metastatic stage (3).

**Figure 1:**
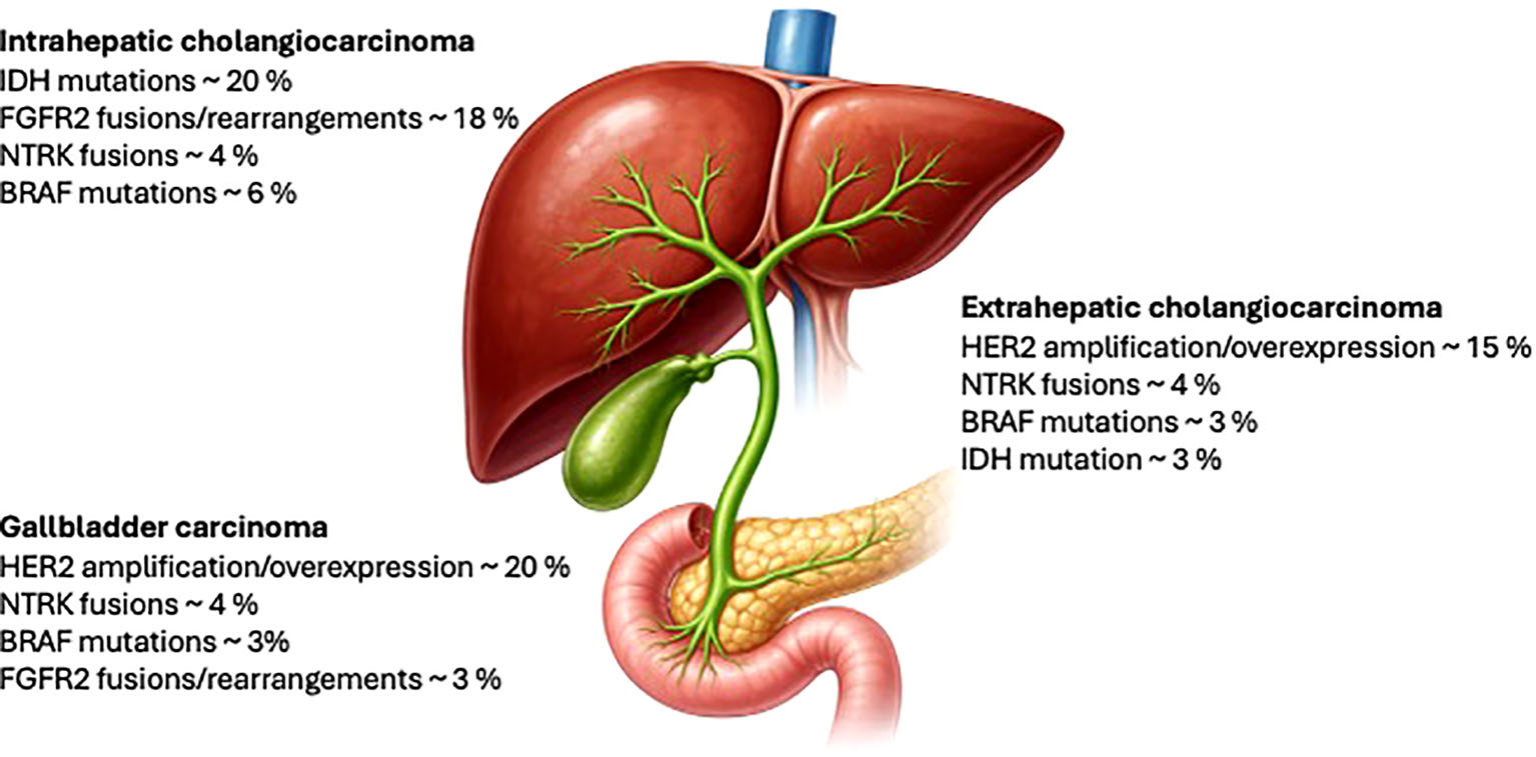
Frequency of mutations according to anatomical location.

Currently, due to advances in molecular biology, there is a general shift in oncology away from a one-size-fits-all approach to a more personalised approach, where therapy is chosen based on the presence or absence of specific predictive and prognostic markers. CCA appears to be a disease rich in potentially targetable alterations. The frequency of individual alterations varies between anatomical locations, with the highest incidence of potentially targetable alterations in iCCA (especially IDH1 and FGFR2). In dCCA, these are mainly BRAF and Her2 alterations (typically in gallbladder tumours) (4, 5). **Figure 1** summarises the most frequent targetable alterations based on anatomic location.

The optimal therapy for CCA is discussed within a multidisciplinary team, with surgery followed by adjuvant therapy being a potentially curative modality in the early stages. In the case of locally advanced and/or metastatic disease, systemic chemotherapy is the basis of therapy. However, there is currently a growing proportion of patients who are treated with targeted therapies according to the presence of identified targetable alterations.

## 2 Non-metastatic disease: surgery and adjuvant therapy

In the early stages of CCA, the treatment of choice is potentially curative surgery followed by adjuvant therapy to reduce the risk of disease recurrence, which is reported in up to 80% of cases within 3 years of resection (6, 7).

BCAT, a randomised phase III study, evaluated the effect of adjuvant gemcitabine versus observation in patients after resection. Only patients with pCCA and dCCA were included in the study. A total of 225 patients were randomised in a 1:1 ratio. The results of the study were negative, and no benefit of adjuvant gemcitabine after resection was demonstrated [median overall survival (mOS) 62.3 vs. 63.8 months, hazard ratio (HR) 1.01, p = 0.964]. No difference was observed in the time to relapse [median relapse-free survival (mRFS) 36.0 vs. 39.9 months, HR 0.93, p = 0.693]. No significant benefit in favour of adjuvant chemotherapy was found also in subgroups stratified according to lymph node involvement and R0 vs. R1 resection. However, only 52.1% completed the planned 6 cycles of adjuvant gemcitabine according to the protocol due to toxicity (8).

The benefit of the combination regimen in the population of patients with metastatic disease led to efforts to test platinum doublet therapy in the adjuvant setting. PRODIGE 12, a randomised phase III study, evaluated the effect of adjuvant gemcitabin + oxaliplatin (GEMOX) vs. observation in patients with CCA regardless of the location of the primary tumour. The primary endpoint of the study was RFS. A total of 196 patients were randomised 1:1. This study also failed to show a benefit in RFS for patients treated with GEMOX vs. observation (30.4 vs. 18.5 months, HR 0.88, p = 0.48). Similarly, no benefit was found in the mOS (75.8 vs. 50.8 months, HR 1.08, p = 0.74). An interesting finding of the study was that adjuvant GEMOX did not have a negative effect on the time to deterioration in patients’ quality of life compared with observation (9).

However, CCA is a highly heterogeneous disease in terms of anatomical location, the occurrence of potentially targetable alterations, R0 vs. R1 resection, and N0 vs. N1 disease. The fact that studies that randomised patients regardless of anatomical location, R0 vs. R1 resection, or lymph node involvement were negative led to an attempt to determine whether the benefit of adjuvant chemotherapy would be demonstrated in a more selected population of patients with CCA.

STAMP was a study that randomised 101 patients with exclusively extrahepatic bile duct tumours (pCCA and dCCA) and N1 disease in a 1:1 ratio between cisplatin (cDDP) + gemcitabine vs. capecitabine. The primary endpoint of the study was disease-free survival (DFS). The 2-year DFS was 38.5% vs. 25.1% (HR 0.96, p = 0.430), and the mOS was 35.7 vs. 35.7 months (HR 1.08, p = 0.404) for cDDP + gemcitabine vs. capecitabine, respectively. As expected, G3–4 toxicity was higher in the combination therapy arm (84% vs. 16%). It can therefore be concluded that this study was also negative and that the combination regimen did not provide any benefit for patients compared with adjuvant capecitabine (10).

Currently, there are only two studies with adjuvant chemotherapy that have demonstrated its benefit compared to observation: the English BILCAP study and the Asian ASCOT study.

BILCAP, a randomised phase III study, randomised 447 patients regardless of anatomical location in a 1:1 ratio to adjuvant capecitabine vs. observation. The primary endpoint was OS. In the intention to treat (ITT) population, the mOS was 51.1 months in the capecitabine arm and 36.4 months in the observation arm (HR 0.81, p = 0.097). In the per-protocol population, the mOS was 53 months in the capecitabine arm and 36 months in the observation arm (HR 0.75, p = 0.028). BILCAP is the first study of adjuvant therapy in CCA to demonstrate a benefit of adjuvant therapy compared with observation (11). The benefit of adjuvant capecitabine was also demonstrated at longer median follow-up (12). The benefit was also demonstrated regardless of R0 vs. R1 resection, N0 vs. N1 disease, and well/moderately vs. poorly differentiated tumours (12).

ASCOT, a randomised phase III study, randomised 440 patients regardless of anatomical location in a 1:1 ratio to adjuvant S-1 vs. observation. The primary endpoint was OS. The 3-year OS was 77.1% vs. 67.6% for the S-1 arm (HR 0.69, p = 0.0080). The 3-year RFS was 62.4% vs. 50.9% in favour of the adjuvant therapy arm (HR 0.80, p = 0.088). The study thus demonstrated the benefit of adjuvant S-1 in patients after resection in the Asian population (13).

The results of the ACTICCA-1 study are currently awaited. ACTICCA-1 is testing adjuvant chemotherapy with cDDP + gemcitabine vs. observation (original protocol), or cDDP + gemcitabine vs. capecitabine, which is the current standard adjuvant therapy for patients with CCA (14).

Currently, phase III, randomised, double-blind, placebo-controlled, multicentre, global study (ARTEMIDE-Biliary01) is ongoing to assess the efficacy and tolerability of rilvegostomig compared to placebo in combination with the investigator’s choice of chemotherapy [capecitabine, S-1 (tegafur/gimeracil/oteracil), or gemcitabine/cisplatin] as adjuvant treatment in participants with biliary tract cancer (BTC) after resection with curative intent. The primary endpoint is recurrence-free survival, and the key secondary endpoint is overall survival. Additional endpoints include progression-free survival following recurrence, patient-reported tolerability, and safety. Enrolment has begun, and approximately 200 sites will be recruiting globally across Asia, Australia, Europe, North America, and South America (15).

Due to the relatively frequent local/locoregional recurrences, the role of radiotherapy in adjuvant therapy for CCA has also been of interest. However, due to the heterogeneity and relative rarity of this type of disease, mostly retrospective data are available. One of the few prospective studies is SWOG 0809, a phase II multicentre study that included 79 patients with extrahepatic CCA and gallbladder cancer. Only patients with T2–4, N1, or R1 resection were included. Patients were treated with 4 cycles of adjuvant combination chemotherapy with gemcitabine + capecitabine, followed by chemoradiotherapy (capecitabine was administered concomitantly). The 2-year OS was 65%, and the mOS was 35 months (16). Despite the limited number of patients, it appears that radiotherapy may be considered in the adjuvant treatment of dCCA, pCCA, and gallbladder cancer, especially in cases of R1 resection.

The results of the individual studies presented on adjuvant therapy in patients with CCA cannot be directly compared (sample size, anatomical location, and European/Asian population), but international guidelines currently recommend adjuvant therapy with capecitabine for the vast majority of patients with CCA after surgery.

## 3 Non-metastatic disease: liver transplantation

Moreover, in selected patients with unresectable, stage I and II perihilar cholangiocarcinoma, treatment with neoadjuvant external beam radiotherapy, brachytherapy, and 5-fluorouracil and/or oral capecitabine (Mayo Clinic protocol) prior to liver transplantation is an option. Initially, 56 patients underwent treatment between 1993 and 2003. Four patients died, and four had disease progression prior to the completion of neoadjuvant therapy. Forty-eight patients underwent operative staging, and 14 had findings precluding transplantation. Twenty-eight patients underwent transplantation, and six were awaiting transplantation. Three patients died from perioperative complications, and four developed recurrent disease 22 to 63 months after transplantation. Actuarial patient survival was 54% at 5 years for all 56 patients, 64% for 48 operatively staged patients, and 84% for 34 patients with negative staging operations. Actuarial survival was 88% at 1 year and 82% at 5 years after transplantation (17).

Updated results confirmed the superiority of liver transplantation versus resection. Specifically, 1-, 3-, and 5-year patient survival rates were 92%, 82%, and 82% after transplantation and 82%, 48%, and 21% after resection, respectively. There were fewer recurrences in the transplant patients (13% versus 27%) (18).

Several clinicopathological predictors of recurrence were identified: older patients and those with high CA 19-9 levels, and larger tumours are more likely to develop recurrent disease (19).

In 2020, an updated study was presented. Between 1993 and 2018, 349 patients were enrolled, with 79% (n = 277) proceeding to the staging operation and 60% (n = 211) undergoing liver transplantation. Intention-to-treat analysis (from the start of neoadjuvant therapy, including patients who did not undergo transplantation) yielded 1-, 5-, and 10-year survival rates of 80%, 51%, and 46%, respectively. Survival rates after transplantation were 91%, 69%, and 62% at 1, 5, and 10 years, respectively (20).

There is also accumulating evidence that liver transplantation can be an option for patients with precisely selected small iCCA. In 2014, a Spanish multicentre retrospective study reported that the 5-year OS following liver transplantation in patients with cirrhosis and small (<2 cm) incidental iCCA was 65% (21). In the follow-up multinational retrospective cohort derived from 17 transplant centres, the 1-, 3-, and 5-year OS rates were 93%, 84%, and 65%, respectively, for 48 transplanted patients with cirrhosis and small (<2 cm) incidental iCCA (22). Liver transplantation recipients also had low tumour recurrence. These outcomes approach those achieved for patients transplanted due to other malignancies, such as HCC, and are superior to those achieved with liver resection.

In 2021, MD Anderson Cancer Center reported on data of 32 patients listed for liver transplantation, and 18 patients underwent liver transplantation. For transplanted patients, the median number of iCCA tumours was 2, and the median cumulative tumour diameter was 10.4 cm. Patients receiving liver transplantation had an overall survival at 1, 3, and 5 years of 100%, 71%, and 57%, respectively. Recurrences occurred in seven patients and were treated with systemic therapy and resection (23). Finally, in 2022, UCLA reported updated outcomes of liver transplantation for iCCA, with 1-, 3-, and 5-year OS rates of 80%, 63%, and 49%, respectively (24).

Recently, a review summarising current recommendations for liver transplant in biliary tract tumours from international societies was published (25).

## 4 Locoregionally advanced disease: neoadjuvant therapy

Due to the asymptomatic early stages, the vast majority of patients with CCA are diagnosed at a locoregionally advanced or metastatic stage (moreover, metastatic spread often occurs despite surgery and adjuvant therapy).

In many cases of locally advanced disease, neoadjuvant therapy is indicated for a number of tumours (breast, pancreatic, and rectal) with the aim of downsizing and downstaging the disease to enable potentially curative surgery. In the case of bile duct tumours, significantly less data are available. This is due to the relatively low incidence of this malignancy, the biological heterogeneity of individual subtypes of cholangiocarcinoma depending on their location in the biliary tree, and the fact that there is no known preferred chemotherapy regimen. Moreover, there are discussions regarding the inclusion of radiotherapy in the algorithm, and finally, there is no consensus on the stage of disease at which neoadjuvant therapy is clearly indicated.

Recently, at American Society of Clinical Oncology (ASCO) 2025, the results of two phase III studies testing a neoadjuvant approach in patients with bile duct tumours were presented. The POLCA-GB study randomised 124 patients with locoregionally advanced gallbladder cancer (T3/T4, N+, and M0 according to staging laparoscopy) in a 1:1 ratio to preoperative chemotherapy (4 cycles of cDDP + gemcitabine) vs. preoperative chemoradiotherapy and chemotherapy (lethal dose (LD) 55–57 Gy to the primary tumour site, or 45 Gy to the regional lymph nodes in 25 fractions + 2 cycles of cDDP + gemcitabine) followed by surgery and the completion of adjuvant chemotherapy (2 cycles of cDDP + gemcitabine in the arm with neoadjuvant chemotherapy and 4 cycles of cDDP + gemcitabine for patients in the arm with neoadjuvant chemoradiotherapy). The primary endpoint of the study was OS; the secondary endpoint was event-free survival (EFS), R0 resection rate, acute and late toxicity, postoperative complications, and quality of life of patients in each arm. The study demonstrated significantly longer survival in the neoadjuvant chemoradiotherapy arm (mOS: 21.8 vs. 10.15 months, p = 0.0028). Neoadjuvant chemoradiotherapy also led to an improvement in EFS (10.61 vs. 4.89 months, p = 0.001). With regard to surgery, 65% of patients in the neoadjuvant chemoradiotherapy arm and 45.7% of patients in the neoadjuvant chemotherapy arm were indicated for exploration (p = 0.02). The R0 resection rate was higher in the neoadjuvant radiotherapy arm (84.2% vs. 70.3%, p = 0.02) (26).

The second study to demonstrate a signal of benefit of neoadjuvant/perioperative chemotherapy in patients with biliary tract tumours (gallbladder cancer, iCCA, and extrahepatic cholangiocarcinoma) was the GAIN study. Patient enrolment in the study was terminated after 68 patients were enrolled due to slow recruitment. Patients were randomised in a 1:1 ratio to neoadjuvant/perioperative chemotherapy (3 cycles of cDDP + gemcitabine before and after surgery) vs. primary surgery +/− adjuvant therapy at the investigator’s discretion. Despite the limited number of patients enrolled, the study demonstrated a clear benefit of the neoadjuvant/perioperative approach, with a mOS of 27.79 vs. 14.62 months, p = 0.0355. Similarly, the benefit of the experimental arm was observed in the EFS parameter (14.62 vs. 4.37 months, p = 0.0032). Neoadjuvant/perioperative treatment also led to a higher percentage of R0 resections and did not lead to an increase in morbidity (27).

Nevertheless, currently in locoregionally advanced disease, the two new trials (POLCA-GB and GAIN) described have several limitations, including small sample size and premature termination. Compared to the BILCAP trial, with a median survival of 51 months in the adjuvant chemotherapy arm, these results for resectable disease are underwhelming.

To improve these results, it is of utmost importance to better define the group of patients with bile duct tumours for whom neoadjuvant therapy is indicated and could lead to improved oncological outcomes.

Another option for improving outcomes for locally advanced iCCA is a combination of radioembolisation and chemotherapy, as shown by Edeline et al. (28). In their phase II clinical trial MISPHEC, they included patients with unresectable, previously not treated iCCA to chemotherapy (cisplatin and gemcitabine) followed by selective internal radiotherapy treatment (SIRT) with yttrium-90 microspheres. The primary endpoint was the response rate at 3 months according to Response Evaluation Criteria in Solid Tumors (RECIST) 1.1. The secondary endpoints were toxic effects, progression-free survival, overall survival, and disease control rate. Of 41 patients included in the study, 26 (63%) were male, with a mean age of 64.0 years. Response rate according to RECIST was 39% at 3 months, according to local review, and was confirmed at 41% as best response by central review; disease control rate was 98%. After a median follow-up of 36 months, median progression-free survival was 14 months, with progression-free survival rates of 55% at 12 months and 30% at 24 months. The median overall survival was 22 months, with overall survival rates of 75% at 12 months and 45% at 24 months. Of 41 patients, 29 (71%) had grades 3 to 4 toxic effects; nine patients (22%) could be downstaged to surgical intervention, with eight (20%) achieving R0 surgical resection. After a median of 46 months after surgery, the median relapse-free survival was not reached among patients who underwent resection (28). Thus, combination chemotherapy and SIRT demonstrated antitumor activity as first-line treatment of unresectable iCCA, and a significant proportion of patients were downstaged to surgical intervention.

## 5 Locoregionally advanced and metastatic disease: without targetable alterations

The standard treatment for patients with locoregionally advanced or metastatic disease is palliative chemotherapy. Since 2010, the standard treatment for patients with metastatic disease has been a combination of cDDP + gemcitabine. The phase III registration study, ABC-02, randomised 410 patients to cDDP + gemcitabine vs. gemcitabine alone. The study demonstrated a significant benefit of the combination regimen, with a mOS of 11.7 vs. 8.2 months (HR 0.64, p < 0.001). A benefit was also demonstrated in the median progression-free survival (mPFS) for the combination regimen of 8.0 vs. 5.0 months (HR 0.63, p < 0.001). Intensification of therapy also led to improved disease control (complete remission (CR) + partial remission (PR) + stable disease (SD)), 81.4% vs. 71.8%, p = 0.049 (29).

Tumours of the hepatobiliary tract generally have a very poor prognosis, and therefore, options for improving these unsatisfactory results have been sought. Analogous to pancreatic cancer, mFOLFIRINOX was tested against the standard cDDP + gemcitabine in patients with locoregionally advanced or metastatic CCA. In a phase II–III study, PRODIGE 38 AMEBICA, this approach was evaluated in a population of 191 patients with CCA. The primary endpoint of the study was 6-month PFS. In patients treated with mFOLFIRINOX, the 6-month PFS was 44.6% vs. 47.3% in patients treated with cDDP + gemcitabine. The mPFS was similarly 6.2 vs. 7.4 months, and the mOS was 11.7 vs. 13.8 months. Regimen intensification did not result in a significant improvement in objective response rate (ORR) (35% vs. 19.4%). Furthermore, subgroup analysis did not identify a patient population that would benefit more from mFOLFIRINOX than cDDP + gemcitabine. However, toxicity ≥ G3 was comparable between the arms (72.8% vs. 72%) (30).

The efficacy of the triplet was also evaluated in a phase II study testing the cDDP + gemcitabine + nab-paclitaxel (GAP) regimen. Among the 60 patients enrolled, very promising results were achieved, with an mPFS of 11.8 months, a mOS of 19.2 months (31), and an ORR of 45%. The very promising results of the GAP regimen led to the initiation of a phase III study, SWOG 1815, which aimed to demonstrate the superiority of GAP vs. cDDP + gemcitabine in a population of patients with locoregionally advanced (27%) or metastatic disease (73%). A total of 441 patients were enrolled in the study with a 2:1 randomisation ratio. However, the results presented did not meet expectations based on the positive signals from the phase II study. The mOS was 14.0 months for GAP vs. 12.7 months for cDDP + gemcitabine, p = 0.65. Similarly, no significant difference was observed in the mPFS (8.2 vs. 6.4 months, p = 0.43). No significant difference was observed in subgroups according to disease location (iCCA, pCCA, and dCCA) or stage (locoregionally advanced and metastatic). However, there was a non-significant trend towards longer mOS in patients with gallbladder tumours (17.0 vs. 9.3 months) and in patients with locoregionally advanced disease (19.2 vs. 13.7 months). However, these are subgroup analyses with a small number of patients, and therefore, these results need to be validated in larger patient cohorts (32). Given the high ORR of the GAP regimen and the promising results in patients with locoregionally advanced disease, further study of this regimen in patients as part of a neoadjuvant approach should be considered.

Currently, therefore, intensification of chemotherapy is not a way to improve outcomes in patients with locoregionally advanced and/or metastatic disease. In addition to intensifying therapy, efforts have been made to improve treatment outcomes by modifying the structure of standard preparations to increase their efficacy. The results of a phase III study, NuTide:121, in untreated patients (n = 773) randomised 1:1 to cDDP + gemcitabine vs. cDDP + NUC-1031 have recently been published. NUC-1031 is a modified gemcitabine with a number of advantageous properties according to preclinical studies (e.g. higher concentration of the active substance inside tumour cells). Promising preclinical signals were not confirmed, and the study was terminated prematurely. Although NUC-1031 + cDDP therapy resulted in a higher ORR compared to the control arm (18.7% vs. 12.4%), this did not translate into an improvement in the primary endpoint of the study, with a mOS of 9.2 vs. 12.6 months (HR 1.79). At the same time, no benefit of the experimental arm was observed in the mPFS parameter (4.9 vs. 6.4 months, HR 1.45). In addition, more patients in the experimental arm had higher rates of liver toxicity, leading to earlier discontinuation of treatment compared to the control arm (33).

In general, bile duct tumours are considered immunologically cold tumours, where there is little expectation of benefit from immunotherapy. However, studies show that bile duct tumours also express PD-L1 and CTLA-4 in the tumour microenvironment (34). Furthermore, it is generally accepted that the combination of chemotherapy and immunotherapy may also be effective in so-called immunologically cold tumours, where chemotherapy increases the expression of neoantigens, leading to synergy with immunotherapy.

This hypothesis was confirmed in a phase II study in which patients with CCA were treated with one of three combinations: cDDP + gemcitabine → cDDP + gemcitabine + durvalumab + tremelimumab (n = 32), cDDP + gemcitabine + durvalumab (n = 49), or cDDP + gemcitabine + durvalumab + tremelimumab (n = 47). The cDDP + gemcitabine + durvalumab arm was associated with an ORR of 72% and a mOS of 20.2 months, leading to the initiation of a phase III registration study, TOPAZ-1 (35, 36).

In the TOPAZ-1 study, 685 patients were randomised in a 1:1 ratio to cDDP + gemcitabine + durvalumab vs. cDDP + gemcitabine + placebo. Patients could be treated with up to 8 cycles of chemoimmunotherapy, and if they are progression-free, they continued on durvalumab monotherapy every 4 weeks until progression. The study met its primary endpoint, demonstrating the superiority of chemoimmunotherapy (mOS 12.8 vs. 11.5 months, HR 0.80, p = 0.021). In addition, during follow-up, there was an increase in the numerical difference in surviving patients in favour of the immunotherapy arm (at 12 months, 54.1% vs. 48%; at 18 months, 35.1% vs. 25.6%; and at 24 months, 24.9% vs. 10.4%). The addition of durvalumab also led to an improvement in the mPFS (7.2 vs. 5.7 months, HR 0.75, p = 0.001). The addition of immunotherapy to standard chemotherapy also led to an increase in ORR (26.7% vs. 18.7%). The vast majority of these were PRs. CR was reported in 2.1% of patients. Subgroup analyses did not clearly identify a population that would benefit more from the addition of durvalumab compared to other subgroups. In some patients, the addition of immunotherapy may lead to long-term disease control; however, we are currently unable to identify this subgroup precisely. With regard to toxicity, it should be noted that the addition of immunotherapy did not lead to a negative impact on G3–4 toxicity (75.7% vs. 77.8%) or to the discontinuation of any component of the regimen (13% vs. 15.2%) (36). The combination of cDDP + gemcitabine + durvalumab is currently the preferred treatment regimen for patients with locoregionally advanced and/or metastatic disease by a number of international oncology societies. In addition, recently published 3-year survival data confirm the long-term benefit of chemotherapy + durvalumab in some patients (3-year OS: 14.6% vs. 6.9%) (37).

A similar study to TOPAZ-1 is KEYNOTE-966, where pembrolizumab was added to the cDDP + gemcitabine regimen. In this study, patients could also receive up to 8 cycles of cDDP + gemcitabine + pembrolizumab, but then continued with the combination of gemcitabine + pembrolizumab until progression (durvalumab monotherapy in the TOPAZ-1 study). The KEYNOTE-966 study also demonstrated the superiority of chemoimmunotherapy for patients with locoregionally advanced and/or metastatic CCA (mOS 12.7 vs. 10.9 months, HR 0.83, p = 0.0034) (38).

Currently, there are no predictive markers that would indicate a higher benefit of chemoimmunotherapy vs. chemotherapy alone, and therefore, combination therapy is offered to all patients who have no contraindications to immunotherapy. Based on current knowledge, there is no clear preference for durvalumab or pembrolizumab in first-line treatment due to very similar HR and mOS in randomised studies.

Biliary tract tumours are generally considered immune cold, and the effect of immunotherapy may therefore be limited. However, anti-VEGF agents are known to have the potential to modify the tumour microenvironment and thus increase sensitivity to concomitant immunotherapy. This approach was tested in a phase II study, IMbrave 151. A total of 162 patients were randomised 1:1 to chemotherapy (cDDP + gemcitabine) + atezolizumab + bevacizumab vs. chemotherapy (cDDP + gemcitabine) + atezolizumab + placebo. The addition of an anti-VEGF therapy to chemoimmunotherapy was not associated with longer mPFS (8.3 vs. 7.9 months, HR 0.67). At the same time, no difference in the mOS was observed (14.9 vs. 14.6 months, HR 0.97). Nevertheless, the addition of anti-VEGF was not associated with a higher toxicity rate (39).

The vast majority of patients with CCA progress during first-line treatment, requiring a change in regimen to stabilise the disease and maintain the existing quality of life. For a long time, no randomised study was available for this patient population. However, in 2021, the results of the ABC-06 study were published, which randomised patients with disease progression on cDDP + gemcitabine to FOLFOX + active symptom control (ASC) vs. ASC. The study of 162 patients demonstrated a benefit of FOLFOX + ASC in OS (6.2 vs. 5.3 months, HR 0.69, p = 0.031). Although the mOS was only slightly longer by 1 month, more patients in the FOLFOX + ASC arm survived 6 months (50.6% vs. 35.5%) and 12 months (25.9% vs. 11.4%). In addition, FOLFOX + ASC did not lead to a deterioration in the quality of life of these patients (40).

Alternatives to FOLFOX in the second line of CCA treatment may be regimens with irinotecan, either conventional or nanoliposomal. However, data on the administration of nanoliposomal irinotecan are controversial. The Asian NIFTY study, a phase IIb trial, tested a combination of 5-FU + leucovorin + nanoliposomal irinotecan (a regimen used in patients with pancreatic cancer) vs. 5-FU + leucovorin in patients with CCA and progression on cDDP + gemcitabine in the first line. A total of 178 patients were randomised in a 1:1 ratio. The primary endpoint was PFS (4.2 vs. 1.7 months, HR 0.61, p = 0.004), which was achieved. The study also achieved the secondary endpoint of OS (8.6 vs. 5.3 months, HR 0.68, p = 0.02) (41). However, the European NALIRICC phase II study did not demonstrate a benefit of 5-FU + leucovorin + nanoliposomal irinotecan vs. 5-FU + leucovorin, and in addition, the combination was more toxic (42). However, a recently published pooled analysis of patients from the NIFTY and NALIRICC studies supports further research into nanoliposomal irinotecan in a population of patients with biliary tract tumours who progressed on cDDP + gemcitabine in the first line of treatment. The analysis demonstrated longer mPFS and ORR in the experimental arm. At the same time, the need for toxicity management was emphasised (more frequent gastrointestinal toxicity in the European population, while haematological toxicity was more frequently observed in the Asian population) (43).

In cases of locally advanced and/or metastatic disease without evidence of targetable alterations, the standard treatment remains a combination of cDDP + gemcitabine, which can be combined with immunotherapy in the absence of contraindications. In the event of disease progression, we have robust data on the efficacy of FOLFOX in the second line, although it should be noted that the clinical benefit for patients is quite marginal. Further systemic therapy should therefore be carefully considered based on the patient’s overall condition, extent of disease, and duration of response to previous lines of treatment.

## 6 Locoregionally advanced and metastatic disease: with targetable alterations

The frequency and representation of individual alterations vary between anatomical locations, with the highest incidence of potentially targetable alterations in iCCA (especially IDH1 and FGFR). In dCCA, these are mainly BRAF and Her2 alterations (typically in gallbladder tumours) (4, 5).

Given its wide availability, testing using next-generation sequencing (NGS) in patients in good overall condition (performance status (PS) 0–1 WHO) should be performed during first-line treatment in order to have the results available in case of disease progression (3). Current European Society for Medical Oncology (ESMO) guidelines recommend personalised therapy based on the presence of identified alterations (IDH1, FGFR2, HER2, BRAF, and deficient mismatch repair (dMMR)) in the event of progression on first-line palliative chemotherapy.

### 6.1 IDH-mutated cholangiocarcinoma

The IDH1 mutation is the most common alteration in iCCA, occurring in 10%–20% of patients. The most common is the IDH1 mutation R132 (4). The IDH1 mutation (an enzyme involved in cell metabolism) leads to the acquisition of the IDH1 enzyme function, which leads to the accumulation of the oncometabolite 2-hydroxyglutarate, which blocks cell differentiation and leads to the initiation of tumorigenesis. Ivosidenib is a molecule that specifically targets the mutated form of the IDH1 enzyme. Ivosidenib was tested in a phase III study, ClardIDHy, in which 185 pretreated patients were randomised in a 2:1 ratio to ivosidenib vs. placebo. The study met its primary endpoint of PFS, with the experimental arm associated with longer mPFS (2.7 vs. 1.4 months, HR 0.37, p < 0.0001). The 6-month PFS was 32% vs. 0%, and the 12-month PFS was 22% vs. 0% in favour of ivosidenib (44). The updated analysis also published the results of the secondary endpoint of the study, OS. There was also a numerical benefit for ivosidenib in the mOS parameter (10.3 vs. 7.5 months, HR 0.79, p = 0.09), but this was not statistically significant due to the high crossover rate (approximately 70% of patients). An analysis of the mOS that took into account the high percentage of patients who switched to ivosidenib after progression on placebo demonstrated a statistically significant benefit for ivosidenib in the mOS parameter (10.3 vs. 5.1 months, HR 0.49, p < 0.001). Ivosidenib can therefore be considered the standard of care for these patients (45). Real-world data confirming the benefit of ivosidenib are also available. In 2023, a cohort of 11 Italian patients treated with ivosidenib in the second to fourth lines was presented. The observed mPFS was 4.4 months, and the mOS was 15 months. Disease control was 63%, including two patients who achieved partial response (46). Treatment with ivosidenib can be considered in all patients in good general condition with the relevant targetable alteration, obviously with the risk of ineffectiveness and early treatment failure. It is therefore necessary to identify predictors of response to ivosidenib beyond the detection of IDH1 mutations.

Currently, trials testing IDH inhibitors in the first-line setting are ongoing. There are preclinical data showing that mIDH1 suppresses key immune-related genes, with reversal of this effect when mIDH1 inhibitors are administered (47). Recently, results from an open-label, phase I study were published. Trial tested LY3410738, mIDH1/2 inhibitor in 119 patients (n = 94 alone, n = 19 in combination with cisplatin and gemcitabine, and n = 6 with durvalumab). No dose-limiting toxicities were observed. An ORR of 5.2% and a disease control rate (DCR) of 56.9% were observed for patients with relapsed/refractory IDH1 or IDH2 mutated cholangiocarcinoma (dose escalation cohort). The combination of LY3410738 plus cisplatin and gemcitabine (dose expansion cohort) demonstrated a response rate of 42.1%, a median duration of response of 8.1 months, and a median progression-free survival of 10.2 months for patients with newly diagnosed IDH-mutant cholangiocarcinoma (48).

Moreover, results of the safety lead-in phase of ivosidenib plus durvalumab and cisplatin/gemcitabine as a first-line therapy in patients with locally advanced, unresectable, or metastatic cholangiocarcinoma with IDH1 mutation were presented recently at ASCO GI 2026. Seven patients with locally advanced or metastatic mIDH1 cholangiocarcinoma were enrolled. Based on results, ivosidenib 500 mg + durvalumab and cisplatin/gemcitabine was confirmed as the recommended dose, and the expansion phase was initiated. The combination demonstrated a safety profile similar to that of durvalumab and cisplatin/gemcitabine. This dose will be evaluated further during the expansion phase, which will enrol approximately 40 patients (49).

### 6.2 FGFR2 fusion in cholangiocarcinoma

FGFR2 alterations (fusions, mutations, and amplifications) are present in approximately 9% of patients with CCA, with a higher frequency in iCCA (50).

Pemigatinib is a potent inhibitor of FGFR 1, 2, and 3. In a phase II study, FIGHT-202, pemigatinib was tested in patients with disease progression on at least one prior line of therapy. The study had three cohorts: 107 patients with FGFR fusions, 20 with other types of FGFR alterations, and 18 without FGFR alterations. The benefit of pemigatinib was demonstrated only in the cohort with FGFR fusions, where the ORR was 35.5%. In this cohort, the mPFS was 6.9 months, and the mOS was 21.1 months (51). Updated, final results confirmed these data (52). In the case of pemigatinib, there are also growing data from real-world clinical practice confirming the results of the registration study. A study from French and Italian centres was presented, in which a total of 72 patients were treated with pemigatinib with an mPFS of 8.7 months, a mOS of 17.1 months, and a toxicity profile consistent with previous experience (53). A similar benefit from treatment with pemigatinib in real-world practice in patients (n = 120) in the USA was recently presented, with an mPFS of 7.4 months (54).

The phase III FIGHT-302 study will further elucidate the role of FGFR inhibitors in biomarker-selected CCA. FIGHT-302, an open-label, randomised, active-controlled, multicentre, global, phase III study, is comparing the efficacy and safety of first-line pemigatinib versus gemcitabine plus cisplatin in patients with advanced CCA with FGFR2 rearrangements. The primary endpoint is progression-free survival; the secondary endpoints are objective response rate, overall survival, duration of response, disease control rate, safety, and quality of life (55).

Infigratinib, an inhibitor targeting FGFR 1, 2, and 3, was tested in a phase II study in a similar patient population to pemigatinib. Given the known efficacy of pemigatinib only in patients with FGFR fusions, only patients with these alterations were enrolled. A total of 122 patients were enrolled in the study. Infigratinib resulted in an ORR of 23.1% and an mPFS of 7.3 months (56).

Pemigatinib and infigratinib are reversible FGFR inhibitors, and as with any other targeted therapy, selective pressure leads to the emergence of resistant cell clones and treatment failure. Irreversible FGFR inhibitors have been synthesised to increase the effectiveness of FGFR inhibition. One such molecule is futibatinib. It is a covalent inhibitor of FGFR 1, 2, 3, and 4 that has been tested in a phase II study, PHOENIX-CCA2, in pretreated patients. A total of 103 patients were enrolled in the study. The ORR was 42%, the mPFS was 9.0 months, and the mOS was 21.7 months. Numerically higher ORR, mPFS, and mOS values may be due to covalent binding and irreversible inhibition of FGFR by futibatinib. In addition, futibatinib appears to be effective even in the case of mutations in the FGFR2 kinase domain, where other FGFR inhibitors have little or no efficacy (57).

A common feature of all non-selective FGFR 1–4 inhibitors is toxicity (especially hyperphosphataemia and diarrhoea) and the development of resistance. One of the new molecules that eliminate these issues is RLY-4008 (lirafugratinib), a highly selective, irreversible FGFR2 inhibitor (58). Its efficacy was tested in a phase I/II ReFocus study, where RLY-4008 (lirafugratinib) was tested on a population of FGFR-naive patients. The study evaluated 38 patients, and the ORR in these untreated patients was 88% (59).

At ASCO GI 2026, updated efficacy and safety data on RLY-4008 (lirafugratinib) were presented. Pivotal cohort of patients (n = 116) with advanced/metastatic CCA harbouring FGFR2 fusions or rearrangements previously treated with ≥1 systemic therapy received 70 mg once daily until disease progression or unacceptable toxicity. An independent review committee assessed the ORR as 47%, and the median duration of response (DOR) was 11.8 months, where 76.2% of responses lasted >6 months. The median PFS was 11.3 months, with a 12-month rate of 49.2%. The median OS was 22.8 months, with a 12-month rate of 74.6%. The DCR was 96.5%. Common grade ≥3 on-target, off-tumour treatment-related adverse events (TRAEs) included palmar–plantar erythrodysesthesia (32.8%) and stomatitis (12.1%). TRAEs led to dose reductions of 75.9%, a dose interruption of 82.8%, and a treatment discontinuation of 4.3%. Thus, RLY-4008 (lirafugratinib) demonstrated clinically meaningful anti-tumour activity, with manageable and tolerable safety in CCA patients with FGFR2 fusions or rearrangements (60). Tinengotinib is another FGFR2 inhibitor that, due to its unique binding capacity, has the ability to inhibit even resistant mutations where other FGFR inhibitors are ineffective. The first results were presented at ESMO 2023, where in a population of 36 patients with FGFR-refractory CCA, the ORR was 31%, and the mPFS was 6.01 months (61).

Recently, updated results were presented. Patients were assigned to one of four cohorts on the basis of FGFR status: cohort A1, FGFR2 fusions with primary FGFR inhibitor resistance; cohort A2, FGFR2 fusions with acquired FGFR inhibitor resistance; cohort B, other FGFR alterations; or cohort C, FGFR wild-type. In total, 55 patients were enrolled and assigned to one of four cohorts (cohort A1, n = 18; cohort A2, n = 11; cohort B, n = 13; and cohort C, n = 13); all received tinengotinib. The median follow-up was 11.3 months. Among 51 patients included in the efficacy evaluable set, the objective response rate was 6.3% (one confirmed partial response) in cohort A1, 30.0% (three confirmed partial responses) in cohort A2, 23.1% (three confirmed partial responses) in cohort B, and 0% in cohort C. Grade 3 treatment-related adverse events included hypertension in 17 (31%) of 55 patients, palmar–plantar erythrodysesthesia syndrome in seven (13%) patients, and stomatitis in six (11%) patients. Grade 4 treatment-related adverse events occurred in two (4%) of 55 patients (increased lipase, n = 1; and posterior reversible encephalopathy syndrome, n = 1); no grade 5 treatment-related adverse events were reported. These findings suggest that tinengotinib may have activity in patients with cholangiocarcinoma with FGFR2 fusions that progressed following FGFR inhibitor therapy. Anti-tumour activity was also observed in patients with other FGFR alterations (62).

### 6.3 BRAF-mutated cholangiocarcinoma

BRAF-mutated CCA occurs in approximately 5% of patients, with a higher incidence in extrahepatic CCA (eCCA) (4). The efficacy of inhibiting BRAF-mutated CCA with a combination of dabrafenib + trametinib was studied in a phase II study, ROAR. The study enrolled 43 patients with BRAF V600E-mutated, previously treated CCA. The study demonstrated an ORR of 47%, an mPFS of 9.0 months and a mOS of 14.0 months (63).

### 6.4 Her2-positive cholangiocarcinoma

Her2-positive CCA occurs in approximately 19% of gallbladder tumours, 17% of eCCA, and 5% of iCCA (4). These are amplifications, overexpression, and mutations.

The evaluation of Her2 can be performed via immunohistochemistry (IHC) testing, fluorescent *in situ* hybridisation (FISH), or NGS. IHC detects Her2 protein overexpression on the cell membrane in tumour tissue. It is semi-quantitative based on staining intensity and percentage of positive cells. Scoring is typically 0, 1+, 2+ (equivocal), or 3+ (positive). IHC3+ generally indicates Her2 positivity; IHC2+ often needs confirmatory testing. FISH (often considered the “gold standard” for confirming Her2 amplification) detects Her2 gene amplification at the DNA level using fluorescent probes and typically uses dual probes, one for Her2 and one for chromosome 17 (control). A high Her2:CEP17 ratio indicates amplification. NGS detects Her2 gene alterations at the DNA level, including amplification, mutations, and copy number changes, as part of multi-gene panels. The majority of studies evaluating anti-Her2 targeted therapy in CCA primarily rely on IHC testing, with FISH confirmation in IHC2+ cases.

In a phase IIa basket study, MyPathway, patients with pretreated CCA and Her2 alteration (amplification, overexpression, or both) were enrolled for treatment with a combination of trastuzumab + pertuzumab. A total of 39 patients were enrolled, with an ORR of 23%, an mPFS of 4.0 months, and a mOS of 10.9 months (64).

Another study evaluating the benefit of anti-Her2 therapy in CCA was the DESTINY-PanTumor02 study, where one of the cohorts was Her2-positive CCA. A total of 267 patients across the spectrum of Her2-positive tumours were enrolled in the study, of whom 41 had CCA. The tested drug was trastuzumab deruxtecan. This study also involved a pretreated population. The ORR achieved was 22% for all CCA patients, with an mPFS of 7.4 months. In addition, the ORR was as high as 56.3% for IHC3+ CCA patients, but 0% for IHC2+ patients (65).

In contrast to breast cancer, where trastuzumab deruxtecan (T-DXd) demonstrates clinically meaningful activity in HER2-low disease, responses in biliary tract cancer appear largely confined to tumours with strong HER2 overexpression (IHC3+) and/or Her2 amplification (66). Several biological factors may account for this difference. First, absolute Her2 receptor density in HER2-low BTC may fall below the threshold required for efficient antibody–drug conjugate (ADC) binding, internalisation, and intracellular payload release (67). While T-DXd has a high drug-to-antibody ratio and a membrane-permeable payload capable of mediating a bystander effect, sufficient initial target engagement remains necessary to achieve therapeutic drug concentrations within the tumour microenvironment. Second, Her2 amplification in breast cancer typically represents a dominant oncogenic driver and is associated with relatively homogeneous expression. In BTC, Her2 alterations are less frequent and often heterogeneous and may not constitute the principal driver of tumour biology, particularly in HER2-low cases (68). Additionally, the dense desmoplastic stroma characteristic of BTC may further limit ADC penetration and distribution. Finally, HER2 scoring systems validated in breast cancer may not directly translate to BTC, and “Her2-low” categories may reflect biologically lower receptor expression in this setting. Collectively, these factors suggest that a higher biological threshold of Her2 expression may be required for T-DXd efficacy in BTC compared with breast cancer. Another anti-Her2 molecule that has been tested in the Her2-positive CCA population is zanidatamab, a bispecific anti-Her2 antibody. Zanidatamab was evaluated in a phase IIb study, HERIZON-BTC-01. The study enrolled 87 pretreated patients (80 in cohort 1, IHC 2+ or 3+; seven in cohort 2, IHC 0 or 1+). The median duration of follow-up was 12.4 months. The confirmed ORR was 41.3%, and the DCR was 68.8%, with an mPFS of 5.5 months and a 9-month OS of 69.9% (all results for cohort 1). The most common treatment-related adverse events (occurring in >10% of patients) were diarrhoea [32 (37%) patients] and infusion-related reactions [29 (33%) patients]. Of the patients, 16 (18%) had grade 3 treatment-related adverse events; the most common were diarrhoea [four (5%) patients] and decreased ejection fraction [three (3%) patients]. There were no grade 4 treatment-related adverse events and no treatment-related deaths (69).

Recently, the final results from HERIZON-BTC-01 were published. The median follow-up was 33.4 months. The ORR was 41.3%, the median duration of response was 14.9 months, and the median OS was 15.5 months (results for cohort 1). Safety profile supported previously published results with diarrhoea, decreased ejection fraction, and anaemia as the most frequent grade 3 TRAEs. There were no treatment-related deaths. Two patients (3%) discontinued zanidatamab due to TRAEs (70).

Other phase II studies in patients with Her2 alteration include the basket trial SGNTUC-019, where the combination of tucatinib + trastuzumab achieved an ORR of 46.7%, an mPFS of 5.5 months, and a 12-month OS of 53.6% (71). The vast majority of studies in Her2-positive CCA tested anti-Her molecules in patients with Her2 overexpression and/or amplification. An exception is the SUMMIT basket trial, phase II, where neratinib was tested in a population of patients with CCA and Her2 mutation. Based on the observed results (ORR 16%, mPFS 2.8 months), it can be concluded that the response to anti-Her2 therapy is higher in Her2-amplified tumours than in Her2-mutated tumours (72).

### 6.5 dMMR/MSI-H cholangiocarcinoma

deficient mismatch repair (dMMR)/microsatelite instability high (MSI-H) CCA is a very rare entity, occurring in approximately 1% of patients (3). The phase II KEYNOTE 158 study enrolled 233 patients across 27 tumour types to receive pembrolizumab monotherapy. A total of 22 CCA patients were enrolled in the study, where pembrolizumab monotherapy resulted in an ORR of 40.9%, an mPFS of 4.2 months, and a mOS of 24.3 months. This is therefore a treatment option for this very rare subgroup of patients with CCA (73).

### 6.6 KRAS-mutated cholangiocarcinoma

The introduction of KRAS inhibitors into the treatment of certain malignancies has led to increased interest in identifying KRAS mutations in a number of other malignancies with the potential for therapeutic intervention. Recently, results from a retrospective, multicentre, pooled cohort study evaluating KRAS allelic variants were published. In total, 5,813 patients had clinical outcome data available, in whom 1,000 *KRAS*-mutated biliary tract cancers were identified. The prevalence of *KRAS* allelic variants was higher in patients with eCCA (36.1%) and pCCA (28.6%) than in those with iCCA (11.8%) and gallbladder carcinoma (7.6%). Thirty-six *KRAS* allelic variants were identified, and the prevalence rates in descending order were G12D (41%), G12V (23%), and Q61H (8%). The variant G12D had the highest mOS of 25.1 months compared with 22.8 months for Q61H and 17.8 months for G12V variants (74). Similar results regarding the frequency of KRAS allelic variants were published by other groups (4, 75).

The emergence of KRAS G12C inhibitors has raised hope that KRAS, previously considered “undruggable”, can now be targeted. Unfortunately, KRAS G12C represents a minority of known KRAS mutations in biliary tract cancer (1.1%) (4). A cohort of 12 patients with previously treated advanced biliary tract cancer was included in the adagrasib (KRYSTAL-1) phase II study, with five responses documented (response rate 41.7%) with a median PFS of 8.6 months and a median overall survival of 15.1 months (76). In the phase I study of another inhibitor, divarasib, a response was seen in one patient with CCA, with stable disease in another four (77).

## 7 Conclusion

Biliary tract tumours are among the malignancies with a very poor prognosis, regardless of stage. Chemotherapy has reached its maximum potential in both the adjuvant and metastatic stages, and even the intensification of therapy has not yielded the expected results. However, it is clear that the introduction of new molecules into the treatment of this disease is associated with prolonged survival after several years. Studies with immunotherapy are proof of this. The future of therapy for this disease lies in early comprehensive testing using NGS, followed by personalised therapy according to the alterations present in patients in good overall condition who have progressed on first-line treatment. Cholangiocarcinoma is thus another disease where a one-size-fits-all approach is unsustainable, and the future lies in precision medicine.
